# Irreversible colorimetric bio-based curcumin bilayer membranes for smart food packaging temperature control applications†

**DOI:** 10.1039/d4ra01411a

**Published:** 2024-03-15

**Authors:** Ariane Pereira, Maria A. Marques, Joaquim Alves, Maria Morais, Joana Figueira, Joana V. Pinto, Felismina T. C. Moreira

**Affiliations:** a CIETI-LabRISE, School of Engineering, Polytechnic Institute 4249-015 Porto Portugal ftm@isep.ipp.pt; b CIETI – School of Engineering, Polytechnic Institute 4249-015 Porto Portugal; c CEB, Centre of Biological Engineering, Minho University 4710-057 Braga Portugal; d LABBELS – Associate Laboratory Guimarães Braga 4710-057 Portugal; e CENIMAT|i3N, Department of Materials Science, School of Science and Technology, NOVA University Lisbon and CEMOP/UNINOVA Caparica Portugal

## Abstract

Research into innovative food safety technologies has led to the development of smart packaging with embedded chemical sensors that can monitor food quality throughout the supply chain. Thermochromic materials (TM), which are able to dynamically change colour in response to temperature fluctuations, have proven to be reliable indicators of food quality in certain environments. Natural colourants such as curcumin are becoming increasingly popular for smart packaging due to their low toxicity, environmental friendliness and ability to change colour. The innovation in this research lies in the production of a bio-based bilayer membrane specifically designed for irreversible temperature monitoring. Membrane A was prepared by dissolving cellulose acetate and curcumin in acetone at room temperature, with glycerol serving as a plasticiser. At the same time, membrane B was carefully formulated by dissolving cellulose acetate and triethanolamine in acetone, with sorbitol as plasticiser. The preparation of these different membranes revealed a remarkable event: a gradual and irreversible colour transition from an initial yellow to a brick-red hue after 24 hours of storage at 25 °C. The chemical structure and morphological analyses of the membranes were performed using several techniques, including FTIR, DSC and SEM. The membrane labels were adhered to aluminium cans and their colorimetric response was observed over a period of 10 days. Minimal colour variations were observed, confirming the reproducibility and stability of the curcumin-based membranes as temperature sensors.

## Abstract 2

Research into innovative food safety technologies has led to the development of smart packaging with embedded chemical sensors that can monitor food quality throughout the supply chain.

## Graphical abstract

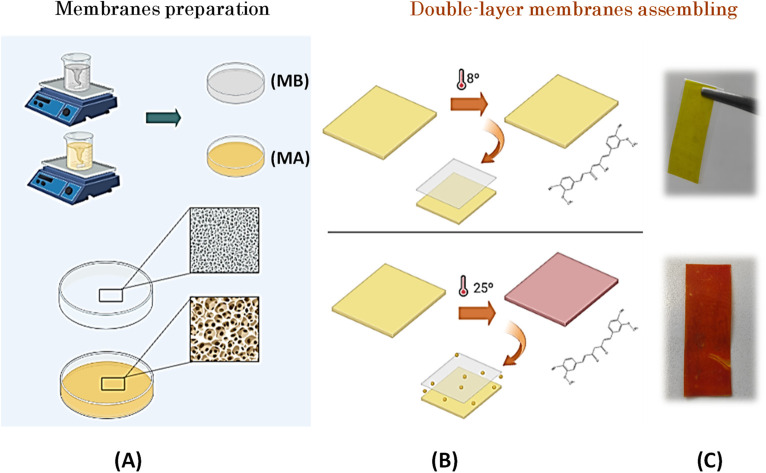

## 1 Introduction

Growing concerns about food safety have led to numerous investigations into novel technologies to regulate food quality.^1,2^ Despite the continued growth in global food production, persistent food shortages remain a major challenge. This problem is exacerbated by the negative impact of food loss and waste throughout the supply chain. More than 30% of edible food is wasted at various points in the production and distribution system.^3,4^ Causes range from food spoilage to oversupply, depending on market conditions or consumer eating and buying behaviours.^5^

Food packaging is of great importance to both industry and consumers as it ensures product safety and quality, facilitates safe transportation and storage, and mitigates loss and damage, thus playing a key role within the overall supply chain.^6^ This is achieved by protecting food from leakage or potential contamination during production, transportation, and storage, thereby extending its shelf life and providing greater convenience to consumers.^7^

The development of smart packaging (SP) represents a promising solution to improve food quality management.^7–11^ This SP development includes both active and smart packaging systems that provide consumers with accurate insight into the condition of the product while offering protection by including elements such as antioxidants, temperature regulators, and antimicrobial agents. This technology facilitates food traceability by carefully assessing and monitoring both internal and external environments, providing information at every point in the food supply chain, from farm to table.^8^ At SP, time–temperature indicators (TTIs) are essential for monitoring food package quality. The management of temperature variations during transportation and storage is critical because they affect microbial proliferation, metabolic processes, and various chemical, sensory, and nutritional changes. The temperature range of 4 to 60 °C, commonly referred to as the “danger zone,” creates favorable conditions for rapid microbial multiplication.^12–14^

Thermochromic materials, a class of smart materials, provide a compelling alternative to temperature sensors because they can change colour in response to temperature changes.^15^ Of particular interest for food quality traceability are materials that exhibit irreversible colour changes and offer the advantage of continuous temperature monitoring throughout a product's shelf life. When the food reaches a critical temperature, the colour change becomes permanent,^16^ leading to greater reliability and reassuring consumers that the proper storage conditions have been met, making the product safe for consumption.

Natural-based thermochromic dyes have emerged as sustainable replacements for synthetic pigments/dyes, primarily due to their minimal to non-existent toxicity, environmental friendliness, ease of manufacture, biodegradability, cost-effectiveness, and occurrence in nature. Natural dyes from compounds such as anthocyanin,^17^ curcumin, betacyanin,^18^ carminic acid, carotenoids, and chlorophylls have the potential to be used in packaging because their natural dye can change under various conditions such as temperature, humidity, pH, and gas evolution.^6^

The current literature contains several examples of biobased thermochromic sensors. For example, researchers successfully developed a colorimetric thermochromic sensor by integrating anthocyanins into a chitosan/cardboard paper film. This innovative sensor completed an irreversible colour change from pale violet to pale yellow within a temperature range of 40 to 70 °C.^19^ In another study, Mataragas *et al.* developed a microbial TTI based on the formation of violacein to monitor the shelf life of vacuum-packed cooked meat products. Violacein is a characteristic purple pigment synthesized by *Janthino bacterium* sp. The hue of the indicator changes from purple to violet as the temperature varies from 0 to 15 °C.^20^ However, an inherent drawback of this technology is that it relies on the presence of *Janthino bacterium* sp. during the initial growth stages to produce violacein and thus facilitate the colour change. Another notable advance is the formulation of a paper-based colorimetric indicator label using natural anthocyanins. Listyarini *et al.*^21^ achieved this by anchoring a natural dye extract from the flowers of Ruellia simplex to cellulose paper through a dip coating process. This indicator was subjected to rigorous testing on shrimp, a perishable food, and stored at different temperatures-13, 25, and 40 °C-for specific periods of time. The observed colour changes included a spectrum ranging from purplish-pink to purplish-blue, then changing to greenish-gray and finally culminating in yellowish-gray.^21^

Another interesting natural dye is curcumin, scientifically known as (1,7-bis(4-hydroxy-3-methoxyphenyl)-1,6-heptadiene-3,5-dione), a polyphenol extracted from the turmeric plant (Curcuma longa).^22,23^ It is usually a mixture of the two primary curcuminoids in Curcuma longa: desmethoxycurcumin and bisdemethoxycurcumin — in a mass ratio of 77 : 17 : 3. In addition to its use as a natural yellow dye, curcumin is also used as a food additive. Curcumin, is known for its antioxidant and antimicrobial properties.^23^ However, it holds importance in food packaging due to its potential to enhance the protective capabilities of packaging materials.^24^ If curcumin is incorporated into packaging, it could help to prevent oxidation and microbial growth and thus extend the shelf life of food. Research into the use of curcumin and other natural compounds in food packaging materials is underway to develop more sustainable packaging solutions.^25^

In this study, we have pioneered the production of an innovative irreversible thermochromic (TC) bilayer membrane for temperature control. This membrane contains curcumin as a dye immobilised in a cellulose acetate (CA) matrix and glycerol, which serves as a plasticiser. A second variant of the membrane was formulated by mixing CA with triethanolamine (TEA) and sorbitol. The membranes prepared in this way showed a gradual colour transition from light yellow to bright red over a period of 24 hours at approximately 25 °C. The choice of CA as a polymer matrix is based on its environmentally friendly properties, its remarkable optical transparency, its cost efficiency, its robust mechanical properties and its high resistance to corrosion strength, and resistance to moisture. These inherent properties make it highly suitable for use in food packaging and highlight its growing importance in the field of biodegradable packaging.^26^

A thorough investigation of the properties of the membranes is a central aspect of our study. We have not only investigated their thermochromic behaviour, but also their morphological, thermal and chemical properties. In addition, we have investigated the potential benefits of this pioneering double-layer membrane in effectively managing temperature fluctuations in the field of intelligent packaging solutions.

From a broad perspective, our research offers a simple, cost-effective and environmentally friendly approach to improving the traceability of sensitive food and pharmaceutical products. The incorporation of curcumin into a cellulose-based bilayer membrane not only emphasises the promise of smart packaging for temperature control, but also has the potential to significantly improve product quality and safety standards. By utilising these bilayer membranes, we aim to make a significant contribution to creating a more robust and reliable framework for protecting the integrity of sensitive food and pharmaceutical products.

## 2 Experimental section

### 2.1 Apparatus

Membranes A and B (MA and MB) and the resulting junction between them were subjected to thermal characterization by differential scanning calorimetry (DSC) using a PerkinElmer system (model DSC 4000) under a nitrogen atmosphere with a gas flow rate of 20 mL min^−1^ and a heating rate of 10 °C min^−1^.

To gain deeper insights into the surface properties of the unique CA membranes, we used scanning electron microscopy (SEM) with the Phenom Pro X system from Phenomworld. A thin layer of gold (10 nm) was carefully applied to the membranes to facilitate imaging. Visualisation was performed at 400× magnification with an electron beam energy of 15 keV.

Chemical characterisation of membranes A and B was performed using a Thermo Scientific Fourier transform infrared spectrometer (Nicolet iS10) equipped with an ATR (attenuated total reflectance) accessory with a diamond crystal. The ATR accessory consisted of a diamond-based support that provided full transparency over the wavenumber range of 400–4000 cm^−1^. The number of scans was set to 150 and the resolution to 8.

For the thermochromic system, which includes the double membrane system, a comprehensive study was carried out at two different temperatures: 8 °C and 25 °C, over a 24 hours period. The temperature of 8 °C was regulated using a refrigerator, while the temperature of 25 °C was maintained in an oven. Images recording the evolving colour change of the bilayer membrane were systematically recorded at different time intervals and under constant illumination conditions. A Nabertherm oven was used to maintain the membranes at a temperature of 25 °C. A self-built darkroom with LED lighting enabled accurate colour control, and the resulting images were captured using a Xiaomi Note 10 Pro smartphone.

### 2.2 Reagents

Curcumin (Sonnentor) and coconut oil (Koala) were purchased from the local market in Porto. Acetone (≥99% – Sigma-Aldrich) was used as a solvent and glycerol (Gly) (≥99% – JMGS), acetate cellulose and sorbitol (≥99% – VWR Chemicals) were used as plasticizers. TEA (≥99% – Sigma-Aldrich) was used to maintain an alkaline pH. All chemical materials and solvents were used without further processing.

#### 2.2.1 Preparation of the thermochromic sensing membrane

**Fig. 1:**
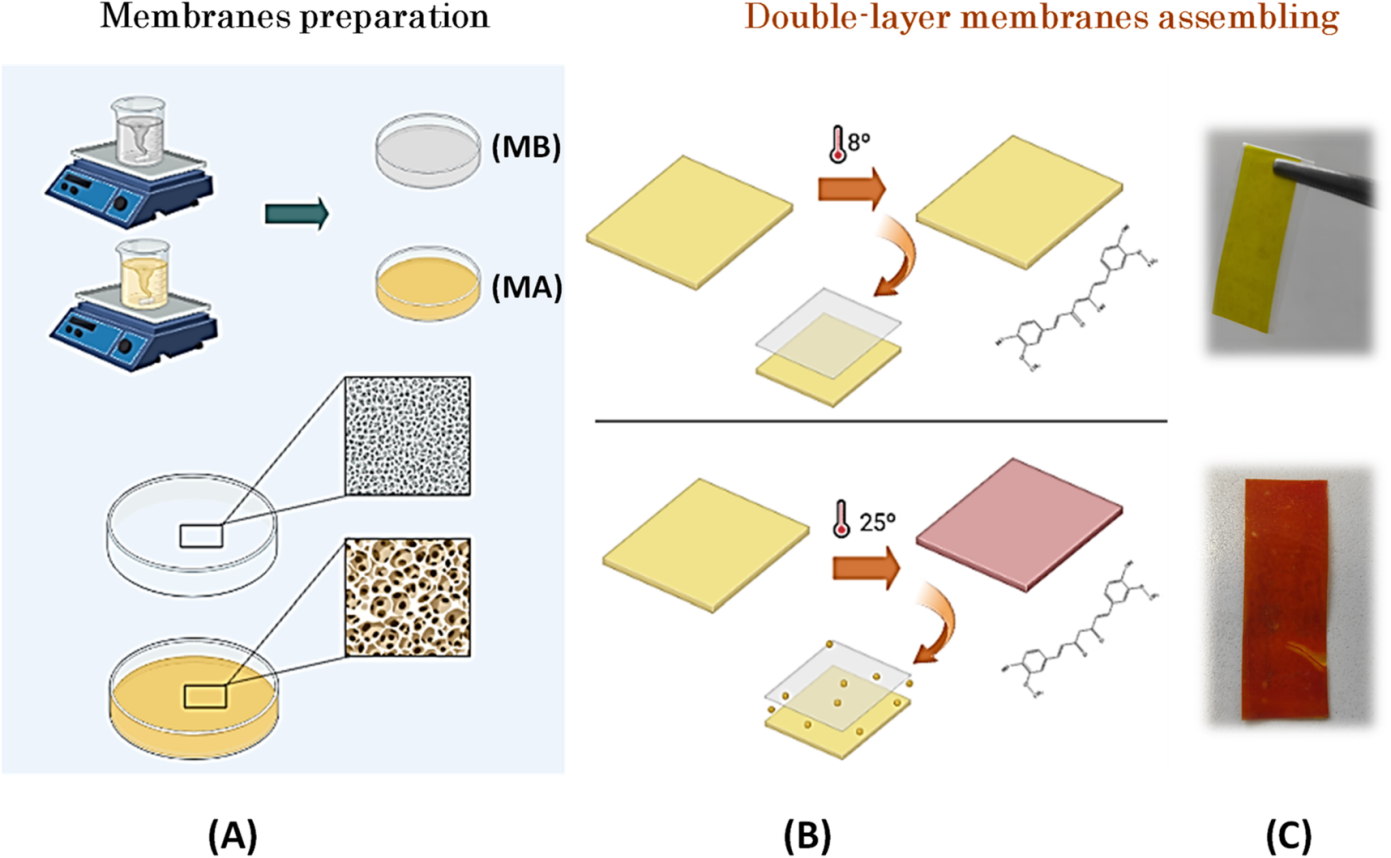
Membranes preparation: double membrane preparation (A); color development after 24 h exposed at 8 °C (upper figures (B) and (C)) and color development after 24 h exposed at 25 °C (lower figures (B) and (C)).

This study was concerned with the development of an innovative double-layer thermochromic membrane. For this purpose, two different membranes named MA and MB (see Fig. 1A and B) were carefully prepared. MA contained curcumin, Gly, and CA (see Fig. S1).† Curcumin was used as a natural dye, while Gly and CA acted as plasticizer and polymeric matrix, respectively. In contrast, MB contained TEA as a dye colour developer in combination with sorbitol as a plasticizer and CA. Acetone was used as a solvent for the preparation of both membranes (see Fig. S1†).

To prepare MA, 1 g CA, 4 g Gly, and 0.02 g curcumin were dissolved in 20 mL acetone at room temperature, and the resulting mixture was magnetically stirred at 1000 rpm for 24 hours. An additional 4 g of Gly was incorporated into the reaction mixture to achieve the desired plasticized consistency. The preparation of MB was carried out in a similar way as that of MA. In this case, 1 g CA was dissolved in acetone in a solution containing 0.1 g sorbitol as plasticizer and 2 g TEA as colour developer with basic character. The reaction mixture was stirred at 550 rpm for 24 hours before being transferred to glass plates. Subsequent homogenization was achieved by stirring at 1000 rpm on a stir plate. The membranes were then stored at room temperature to facilitate evaporation of the solvent. In the final step, the membranes were assembled to allow diffusion of TEA from MB to MA.

#### 2.2.2 Temperature time indicator (TTI)

Each membrane was cut into rectangular dimensions and then assembled. For a more reliable evaluation, the bilayer membranes were then placed in empty aluminium cans, which allowed a more detailed study of the kinetics of colour development in a practical environment. All analyses were performed at a constant temperature of 25 °C, and the subsequent colour change was carefully observed over a 24 hours period and documented using a smartphone.

#### 2.2.3 Specificity tests

To determine whether the irreversible colour change occurs exclusively at 25 °C, a careful examination of the colour change at 8 °C was carried out. Images were taken after 1 hour and 24 hours of exposure and carefully documented the progression of colour change over time. A thin layer of coconut oil was placed between the bilayer membranes to create a barrier against water penetration and thus increase the effectiveness of the membranes.

## 3 Results and discussion

### 3.1 Double-layer membrane preparation

The developed biobased thermochromic sensor is based on a two-layer membrane architecture. The first layer, called MA, consists of a mixture of AC, curcumin, Gly, and acetone. Curcumin, extracted from the rhizomes of Curcuma longa Linn, is a naturally occurring polyphenolic component known for its acceptance as a permissible natural colorant in various applications such as food, nutrition, and pharmaceutical formulations.^26,27^ At the chemical level, curcumin corresponds to (1E,6E)-1,7-bis(4-hydroxy-3-methoxyphenyl)hepta-1,6-diene-3,5-dione. This compound is characterized by three acidic protons with respective p*K*_a_ values of 7.8, 8.5, and 9.0.^28^ Curcumin is insoluble in water under acidic or neutral conditions but dissolves under alkaline conditions.^29^ Gly is a transparent and viscous liquid that dissolves readily in water and other polar solvents. Its distinct polar nature is due to the presence of hydroxyl groups (–OH) in its chemical structure, which facilitate the formation of hydrogen bonds with water molecules and other polar constituents.

These characteristic properties of curcumin and Gly were used to impart the desired properties to the bilayer membrane forming the thermochromic sensor, which is consistent with the observations of Sharma & Pathak.^30^ Due to its inherently polar nature, Gly has the exceptional ability to dissolve curcumin through its interactions with the polar functional groups of the compound. This symbiotic interaction aids the dispersion of curcumin and allows it to assume a soluble form, making Gly an indispensable component of the composition of MA in this particular context. Moreover, Gly not only helps to improve the solubility of curcumin, but also increases the resistance of the membrane to external agents. Gly, in fact, plays a crucial role in improving the mechanical properties not only of CA membranes but also of other biopolymers used in the formulation of packaging films. The addition of plasticizers such as Gly helps to make the final product more flexible and consequently increase its overall performance.^31^

**Fig. 2:**
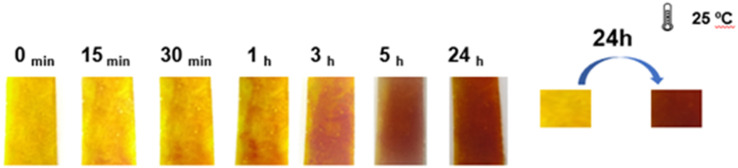
Colour variation over time of the dual membrane system assembled with sticky tape.

To initiate the colour change within the bilayer membrane, the curcumin contained in MA must assembled with the TEA contained in MB. This interaction is facilitated primarily by the addition of Gly. To achieve this cooperative effect, the formulation of MB, which was dissolved in acetone, combined TEA—an alkaline solvent known to cause colour evolution from yellow to brick brown—and sorbitol, which serves as a plasticizer. The incorporation of sorbitol into cellulose membranes is strategic because it brings a number of beneficial properties, including enhanced mechanical properties, increased flexibility, improved resistance to external agents, and the ability to release active components, making these membranes versatile and suitable for numerous applications.^32^ Based on these formulation considerations, the resulting reaction mixture, which contained both MA and MB, was carefully stirred before being transferred to glass plates where the solvent evaporated. The resulting membranes were stored at room temperature (Fig. 2A).

To complete the sensing process, membranes were assembled to allow controlled diffusion of TEA from MB through the membrane barrier to MA (see Fig. 1B and C). After 24 hours of storage in an oven at 25 °C, the bilayer membrane showed a colour change from an initial yellow to a distinct brick red. This transformative effect explains when the membranes are exposed to a temperature of 25 °C, at which TEA undergoes a phase transition and changes from its solid state to a liquid form. This colour change is expectable because according to the literature^22,24^ with the increase in pH from 7 to 8 (alkaline conditions), there is a strong change from yellow to red. This change occurs because the phenolic hydroxyl group reacts easily with OH– to form the phenoxide anion and causes a colour change.

#### 3.1.1 Time–temperature indicator (TTI) study

##### 3.1.1.1 Membranes assembled with sticky tape

The membranes were prepared by the casting method and then joined with adhesive tape, which allowed the controlled flow of compounds from MB to MA and triggered their interaction and thus the change in colour at 25 °C. The progression of colour development was observed over a period of 15 minutes, 30 minutes, 1 hour, 2 hours, 3 hours, 5 hours, and finally 24 hours after assembly and exposure to 25 °C. As can be seen in Fig. 2, the colour change was clearly observed after 30 minutes and as early as 15 minutes, respectively, with the luminance conditions remaining constant throughout the observation period. After 24 hours, the colour changed from a strong yellow hue to a rich brick red.

It is notable that this observable colour change occurred primarily when the bilayer membrane was exposed to temperatures above 25 °C. Throughout the 24 hours exposure period, the membrane showed a homogeneous colour change, with the brick-red colour remaining stable beyond this time window (see Fig. 2).

Overall, these results demonstrate the possibility of tracing and monitoring the exposure of any material to temperatures above 25 °C over a 24 hours period.

##### 3.1.1.2 Effect of the coconut oil as a barrier agent

###### 3.1.1.2.1 Specificity study

**Fig. 3:**
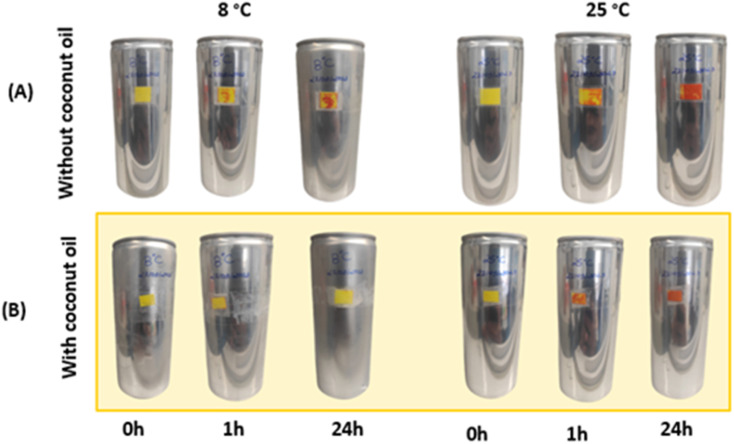
Aluminum cans with double layer membrane made by the blade coating technique. (A) Cans with double layer membrane. (B) Cans with coconut oil barrier between the membranes.

The membranes were attached to empty aluminium cans to test their suitability as temperature sensors in the food industry. Surprisingly, they showed a different thermochromic behaviour under these conditions. In particular, compared to the free-standing membranes described in section 3.1.1, there was a pronounced divergence in the colouration after both 1 hour and 24 hours. This suggests that the underlying surface to which the membranes were joined significantly influences the kinetics of colour development. However, it should be emphasised that this surface-related effect did not alter the basic chemical reaction, as the brick-red colouration was still observed after 24 hours at 25 °C. Fig. 3 shows the results of the double-layer membrane produced using the drop-casting technique and applied to aluminium cans. In this Fig. 3, a clear change in colour from yellow to brick red can be seen, which is a clear indication of the effect of heating the samples at 25 °C. However, it can be seen from Fig. 3A that the membranes already show a colour change at a temperature of 8 °C.

To prevent colour changes at 8 °C, a protective layer of coconut oil was strategically inserted between the membranes. Coconut oil has a melting point of 24 °C, which means that the oil assumes a solid state when the cans are stored in a refrigerated environment, thus serving as a barrier. Conversely, the coconut oil changes to a liquid state once the thermochromic system is removed from refrigeration. The results of this approach, shown in Fig. 3B, indicate that the bilayer membrane shows no colour change when stored at 8 °C, this event is attributed to the effectiveness of coconut oil as a barrier to the flux of species between membranes.

### 3.2 Thermal, chemical and morphological analysis

#### 3.2.1 Scanning electron microscopy (SEM)

**Fig. 4:**
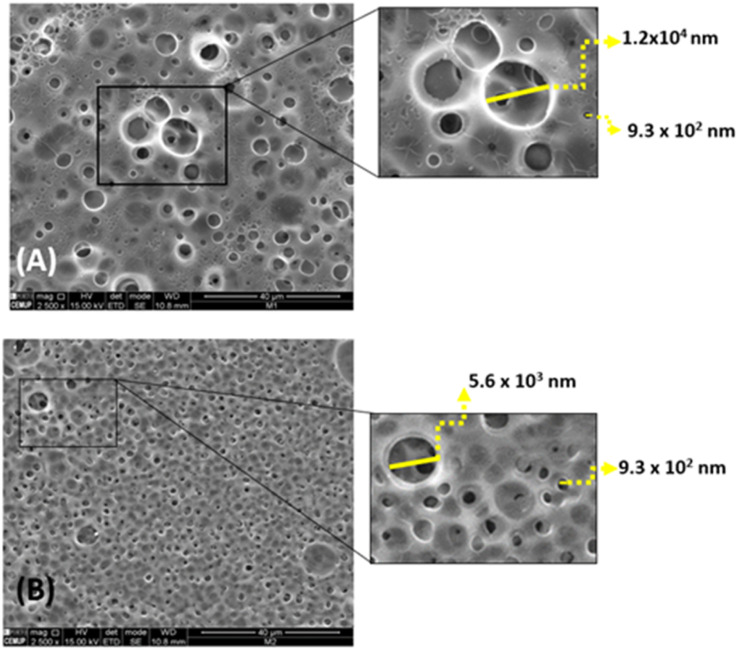
SEM analysis of the (A) MA and MB (B).

Morphological analysis using SEM provides high-resolution three-dimensional images. In this study, both membranes A and B were analyzed to differentiate their porosities. Fig. 4 illustrates that both membranes A and B contain pores. MA exhibits pore sizes ranging from 9.5 × 10^2^ nm to 1.1 × 10^4^ nm, while MB exhibits pore sizes ranging from 6.6 × 10^2^ nm to 5.1 × 10^3^ nm.

The difference in porosity can be attributed to the different plasticisers used for each membrane. MA has sorbitol as plasticiser, while MB uses glycerol. The lower porosity of MB resulting from the presence of sorbitol and TEA can be attributed to the lower affinity of sorbitol to water associated with its strong molecular interactions with the CA chains, enhances the cross-linking between the membrane components.^32,33^

#### 3.2.2 Fourier-transform infrared spectroscopy (FTIR)

**Fig. 5:**
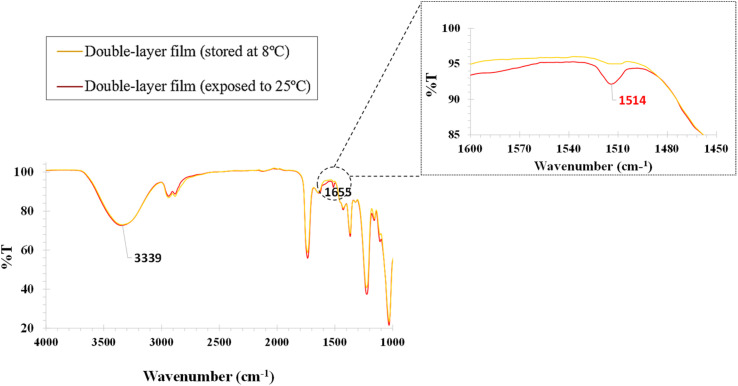
FTIR analysis of double membrane layer exposed at 8 and 25 °C.

FTIR is an analytical technique for identifying and analyzing chemical compounds based on their infrared energy absorption characteristics (Fig. 5). It measures the interaction between molecules and infrared radiation to obtain information about the chemical bonds present in the substance. The FTIR spectra of MA and MB are shown in Fig. 5. The spectral peaks at 3340 cm^−1^ and 1655 cm^−1^ were assigned to the free vibrations of O–H and C–H, respectively, and the peaks at 1225 cm^−1^ and 1030 cm^−1^ were caused by the aromatic stretching of C–O and C–O–C, respectively, which is consistent with the results in the literature.^34^ As you can see, the particular peak corresponding to RRC=O at 1513 cm^−1^ ^35^ present in IR of the bilayer membrane after exposure at 25 °C proves that a change has occurred. When the curcumin particles are transported by Gly and come into contact with TEA, which has an alkaline pH, the peak at 1513 cm^−1^ is associated with the curcumin in its enolate or form under alkaline conditions, in this case a phenoxide anion. Phenoxide anions also exhibit characteristic peaks in the 1600–1400 cm^−1^ range. This shows the transport of curcumin through MA to MB, which is chemically altered on contact with liquid TEA (25 °C) (Fig. 5).

#### 3.2.3 Differential scanning calorimetric (DSC)

To analyze the thermal properties of the fabricated membranes, DSC measurements were performed in air from 0 to 500 °C. The DSC curve of the membrane containing cellulose acetate, Gly, and acetone is shown in Fig. S1.† Four endothermic peaks at 174, 230, 235, and 264 °C can be seen. The endothermic peaks at 100 and 220 °C were attributed to the esterification enthalpy and thermochemical transition (where the acetyl bond is broken).^36^ The loss of mass of cellulose acetate membranes at temperatures up to 200 °C is due to the deacetylation of this polymer. The thermal decomposition of this material involves two phases. The first phase, in which the mass loss is higher, occurs at temperatures between 300 and 400 °C. In this phase, the glycosidic bonds are broken, so that the cellulose acetate molecules decompose into volatile and dehydrated compounds. The second phase, in which the charring of the sample takes place, occurs at temperatures between 400 and 600 °C.^37^

The curve of the membrane containing curcumin, cellulose acetate, Gly, and acetone is shown in Fig. S2,† where 2 endothermic peaks at 172 and 253 °C can be seen. The melting point of curcumin is between 173 and 187 °C. Therefore, the endothermic peak at 172 °C represents the melting point of this compound.^25,38,39^ The DSC curve of curcumin membrane after fusion with triethanolamine is shown in Fig. S2.† Endothermic peaks at 132, 158, 164, 252, and 262 °C can be seen.

The DSC curve of the membrane containing cellulose acetate, sorbitol and acetone is shown in Fig. S3.† It shows an endothermic peak at 235 °C and two exothermic peaks at 342 and 374 °C. It has been reported that the decomposition of d-sorbitol starts at 200 °C.^40^ The DSC data of the membrane containing triethanolamine, cellulose acetate, sorbitol, and acetone are shown in Fig. S2,† where 3 endothermic peaks at 127, 206, and 274 °C and 1 exothermic peak at 337 °C can be seen, which could be related to the boiling of triethanolamine, a phenomenon that occurs at 335 °C.^41^

### 3.3 Specificity studies in water filled cans

**Fig. 6:**
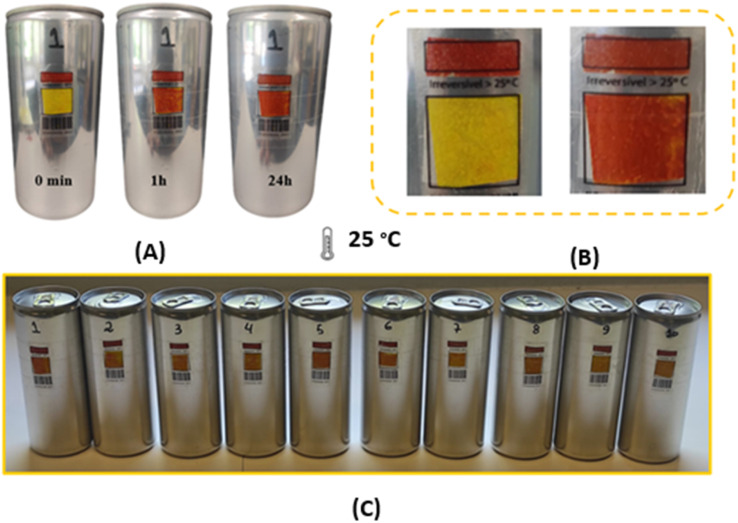
(A) Thermochromic analysis at 25 °C of double-layer membrane applied on filled aluminium cans. (B) Acetate labels with double-layer membrane applied on cans. (C) 10 samples of labels applied on cans, after 1 h of exposure at 25 °C.

Double-layered membranes were attached to aluminium cans with and without water to evaluate their effectiveness in monitoring the temperature of beverages in these containers. Tests were performed on ten samples for a thorough evaluation (see Fig. 6C). The double-layer membrane was adhered to acetate labels, which were then attached to the cans. There was a small red rectangle on the top of the labels that served as a control. It is important to note that the application of the double-layer membrane was directed towards the lower rectangle (see Fig. 6B).

Each individual sample showed a detectable colour change after both a 1 hour and a 24 hours exposure, as shown in Fig. 6A, both for the doses with and without water. In particular, the doses with water showed a more pronounced colour change, which can be attributed to the higher thermal conductivity of liquids compared to gases. Consequently, the cans with water showed a more pronounced and clearer colour transition than their “empty” counterparts. This comprehensive test confirms the suitability of the double membrane system for monitoring products that need to be stored in a temperature range of 5 °C to 8 °C.

## 4 Conclusion

The production and comprehensive analysis of the bio-based curcumin bilayer membrane developed for temperature monitoring has been successful. The bilayer membrane, which consists of both MA and MB components, has shown a colour change from yellow to red at a temperature of 25 °C. Remarkably, this colour change is due to the supporting role of Gly, which acts as a vehicle for the seamless transfer of curcumin particles from MA to MB and leads to a modulation of the keto–enol balance.

Notably, the strategic integration of a coconut oil barrier between the membranes prevented undesirable colour changes at 8 °C and improved sensor specificity for temperatures above 25 °C. Remarkably, this colour change is due to the facilitating role of Gly, which acts as a vehicle for the seamless transfer of curcumin particles from MA to MB, resulting in modulation of the irreversible colour change only after 25 °C. This is due to the solidified state of the oil acting as a reliable barrier, while its liquefaction allows smooth migration of Gly and curcumin particles and mixing with the TEA.

The inclusion of DSC measurements enriched our understanding of the thermal properties of the membranes produced, including the melting points of curcumin and the extent of degradation of cellulose acetate and sorbitol, as well as their resistance under extreme conditions and the maximum temperature they can withstand. In addition, empirical analyses carried out on real samples have conclusively demonstrated the effectiveness of the double-layer membrane when applied to aluminium cans for temperature monitoring. The colour changes were clear for cans with and without water exposed to different temperatures, with the greater colour change for the first can being related to the higher thermal conductivity of liquids. The successful performance of the tests with the double-layer membrane, which was applied to acetate labels and attached to aluminium cans, proves that they have a promising future as cost-effective and easy-to-manufacture temperature sensors that can be used in the food or pharmaceutical industry.

Overall, the innovative thermochromic double-layer membrane is very promising for temperature monitoring in various applications, especially in food packaging and storage. These visual indicators provide invaluable insight into temperature conditions and the possible onset of spoilage. Future advances should include further research and refinements that optimise the properties of the bilayer membrane and application methods to increase its effectiveness and commercial utility.

## Conflicts of interest

There are no conflicts to declare.

## Supplementary Material

RA-014-D4RA01411A-s001
