# Supplementary material for: Irreversible colorimetric bio-based curcumin bilayer membranes for smart food packaging temperature control applications
Source: RSC Adv. 2024 Mar 15;14(13):8981–9. doi: 10.1039/d4ra01411a (PMC10941329; doi:10.1039/d4ra01411a)
Supplement: RA-014-D4RA01411A-s001 [file RA-014-D4RA01411A-s001.pdf]

Electronic Supplementary Material (ESI) for RSC Advances.
This journal is © The Royal Society of Chemistry 2024

Supplementary information

Irreversible coulorimetric bio-based curcumin bilayer membrane for Smart Food Packaging temperature control applications

Ariane Pereira[a] , Maria A. Marques[b], Joaquim Alves[b], Maria Morais[e, f], Joana Figueira [e, f], Joana V. Pinto [e, f,] Felismina T.C. Moreira [a,c,d*]

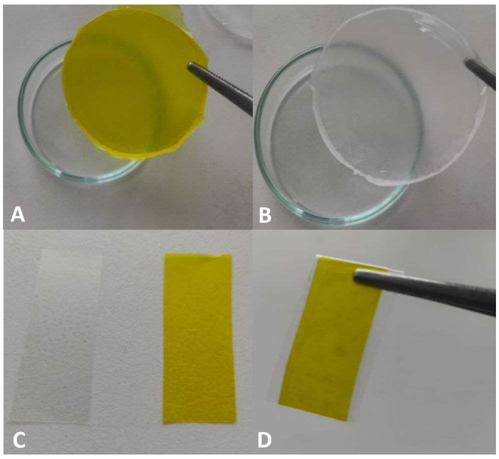

Figure S1 – Double layer membrane preparation. A) Curcumin MA1; B) TEA MB; c) membrane 1 and membrane 2 Double-layer membrane .

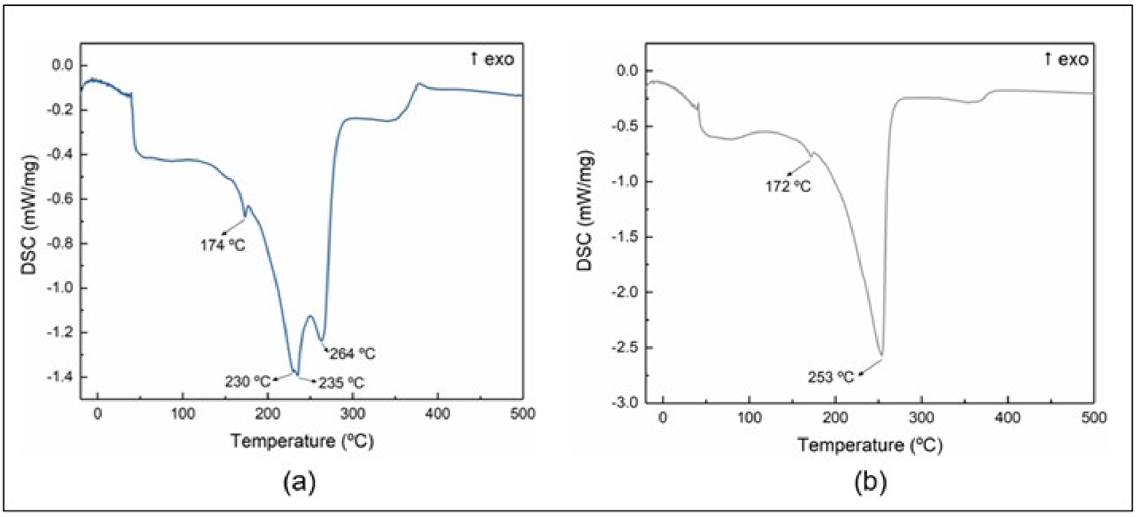

Figure S2 - (a)DSC curve of the membrane with cellulose acetate and glycerol.(b)DSC curve of the membrane with cellulose acetate and curcumin.

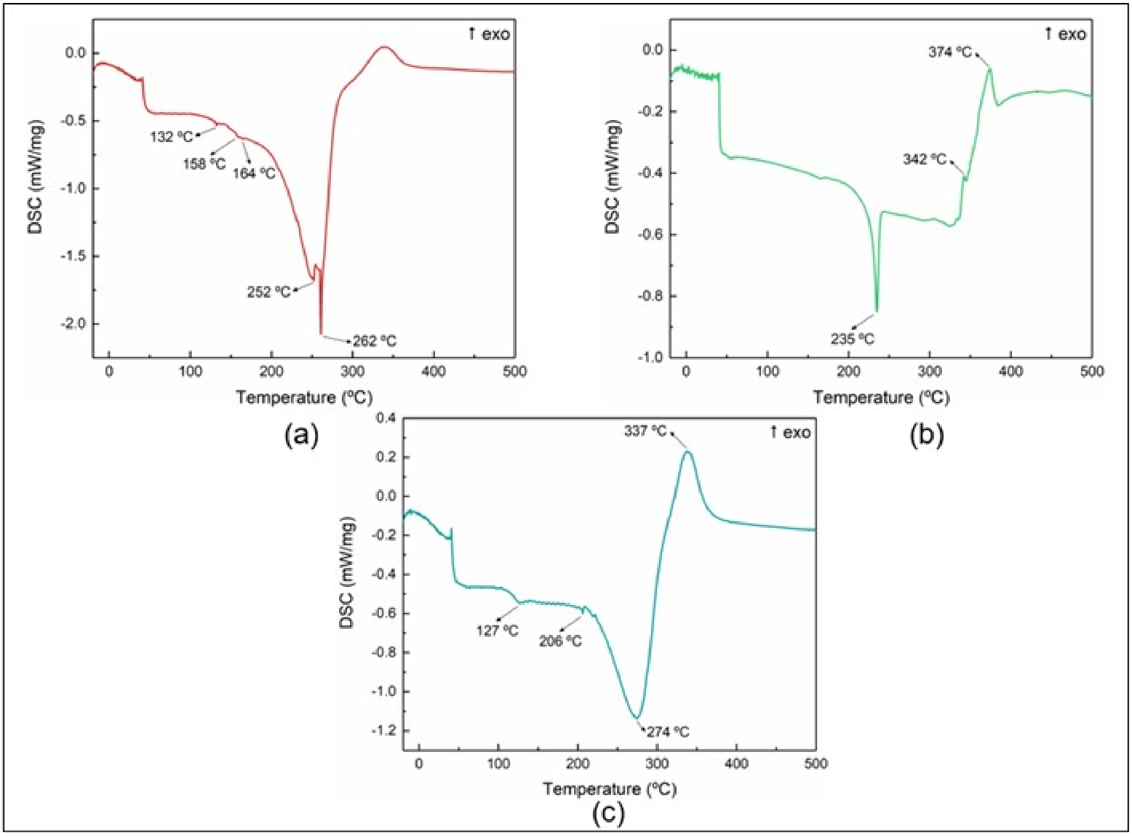

Figure S3- (a)DSC curve of the membrane with curcumin after the reaction with triethanolamine.(b)DSC curve of the membrane with cellulose acetate and sorbitol.(c)DSC curve of the membrane with triethanolamine and cellulose acetate.
